# Response to the Letter to the Editor on “Comparison of Disease Patterns and Outcomes Between Non-Japanese and Japanese Patients at a Single Tertiary Emergency Care Center in Japan”

**DOI:** 10.2188/jea.JE20210435

**Published:** 2022-02-05

**Authors:** Euma Ishii, Nobutoshi Nawa, Hiroki Matsui, Yasuhiro Otomo, Takeo Fujiwara

**Affiliations:** 1 Department of Global Health Promotion, Tokyo Medical and Dental University, Tokyo, Japan; 2 Department of Medical Education Research and Development, Tokyo Medical and Dental University, Tokyo, Japan; 3 Department of Acute Critical Care and Disaster Medicine, Tokyo Medical and Dental University, Tokyo, Japan

We are grateful to the editors for this opportunity to respond to Saeki et al’s^1^ insightful comments concerning our article, “Comparison of disease patterns and outcomes between non-Japanese and Japanese patients at a single tertiary emergency care center in Japan”.^2^ In their letter, Saeki et al discuss the importance of differentiating non-national patients into visitors or residents and called for standardization in data collection throughout Japan that incorporates this differentiation. We applaud Saeki et al for investigating and highlighting this predicament further by exploring the feasibility of using patient addresses and insurance information from their facility to group patients into residents or visitors.

Due to the current lack of a standardized method to query patients about their nationality and background (eg, residents or tourists in Japan), we were unable to incorporate this essential component, which was a limitation to our study. Factors, such as the existence of patients with Japanese names who cannot speak Japanese or non-Japanese patients who are fluent and understand Japanese culture, should have also been considered.

We are in complete consensus with Saeki et al that patient visiting status should be made obtainable from official documents, including residence cards or visa types. In order to realize this, and after careful consideration and discussions with ethics and legal experts concerning the protection of personal information, it may be necessary to work with electronic health record companies as well as non-profit organizations that regulate guidelines over non-Japanese patient care, such as the Japan Medical Service Accreditation for International Patients,^3^ alongside advocacy efforts that call for such policy changes. We hope that the standardization in acquiring patient background information would not only aid hospitals to better prepare resources to overcome language and cultural barriers, but also decrease any hesitancy around non-Japanese patient care while permitting effective allocation of hospital operational budget and government funding in clinical care.
